# Uncertainty-Driven Global-Local Mamba with UNet for gastric cancer histopathological image classification

**DOI:** 10.3389/fphys.2026.1805679

**Published:** 2026-03-26

**Authors:** GeZhiChen Li

**Affiliations:** School of Computer Science and Artificial Intelligence, Shandong Normal University, Jinan, China

**Keywords:** gastric cancer, global-local feature fusion, image classification, UNet, Vision Mamba

## Abstract

Histopathological image classification is important for gastric cancer diagnosis. Existing methods have difficulties in balancing global context modeling and local texture extraction. A novel Uncertainty-Driven Global-Local Mamba with UNet to proposed to solve these problems. It is used for gastric cancer histopathological image classification. The new model has three key innovations. First it has an uncertainty-driven scanning mechanism. This mechanism adjusts the attention weights of global and local feature extraction dynamically. It is based on the confidence of intermediate features and can highlight suspicious lesion regions effectively. It can also suppress irrelevant background noise. Second it has a global-local Mamba module. This module combines bidirectional selective scanning and depthwise separable convolution. Bidirectional selective scanning is used for long-range dependency modeling. Depthwise separable convolution is used for local texture enhancement. Compared with transformer-based methods it achieves linear computational complexity. Third it has UNet-inspired skip connections. These connections fuse multi-scale features from the encoder and decoder. Extensive experiments are conducted on the GasHisSDB dataset. The new approach achieves excellent performance. Its accuracy is 98.76%. Its precision is 98.53%. Its recall is 98.31%. Its F1-score is 98.42%. The new method provides a reliable and efficient tool for computer-aided diagnosis of gastric cancer. It has potential for clinical deployment in pathological analysis workflows.

## 1 Introduction

Gastric cancer (GC) is one of the most common malignant tumors in the world. About 1.1 million new cases and 769,000 deaths are reported every year (Mamun et al., 2024). One out of ten people of the same age born between 2008 and 2017 around the world will get stomach cancer in their lifetime. What is more worrying is that three quarters of these cases could have been prevented. Eradicating Helicobacter pylori could have avoided them (Park et al., 2025). Histopathological examination of hematoxylin and eosin (H&E)-stained tissue sections is known as the gold standard for GC diagnosis (Krishnan, 2023). It allows pathologists to observe cellular morphology, glandular structure and tissue infiltration characteristics. These observations help distinguish benign and malignant lesions. However manual histopathological diagnosis has great challenges in clinical practice. First human judgment is subjective. This leads to inconsistent diagnostic results among different pathologists. This is especially true for early GC with slight pathological changes. Second there are a large number of pathological slides. This brings heavy workloads to pathologists. Fatigue increases the risk of missed diagnosis or misdiagnosis. Third underdeveloped regions lack senior pathologists. This further limits people’s access to high-quality diagnostic services.

To address these issues, the Computer-aided diagnosis (CAD) systems based on deep learning have become a promising solution. They can improve the efficiency and accuracy of GC histopathological analysis. In recent years, deep learning models have made remarkable progress in medical image classification tasks. These models include convolutional neural networks (CNNs) (He et al., 2017; Ronneberger et al., 2015; Zhou et al., 2019; Chen et al., 2019). They also include transformers (Dosovitskiy et al., 2020; Liu et al., 2021; Wang et al., 2021; Heo et al., 2021; Wu et al., 2020; Fan et al., 2021) and vision Mamba (VM) (Zhu et al., 2024). However, existing methods still have limitations when used for GC histopathological image classification. CNNs are good at extracting local texture features through convolutional kernels. But their receptive field is limited. This makes it hard to capture long-range dependencies. Transformers use self-attention mechanisms to model global contextual information. But their computational complexity increases quadratically with the input sequence length. This leads to high memory consumption and slow inference speed. It is incompatible with the large-scale pathological image data in clinical practice. Traditional vision Mamba models use selective state space model (SSM) (Gu and Dao, 2023). They achieve linear complexity in global dependency modeling. But they lack adaptive coordination between global context and local texture extraction. They also fail to consider the uncertainty of feature representation. This results in insufficient robustness to the heterogeneity of GC histopathological images. To note that GC histopathological images have unique characteristics. For instance, both local discriminative features and global structural features exist. Therefore, a new model needs to be developed. This new model should balance global-local feature fusion, computational efficiency and uncertainty-aware feature learning.

To address the aforementioned limitations, this study proposes an uncertainty-driven (UD) Global-Local Mamba (GMamba) with UNet structure for GC histopathological image classification, with the following key contributions:

- A novel UD-GMamba-UNet architecture is proposed, which integrates the UD mechanism, a GMamba module, and UNet-inspired skip connections. This design specifically adapts to the heterogeneity of GC histopathological images, balancing global context modeling, local texture extraction, and fine-grained feature preservation.
- A GMamba module is constructed to achieve efficient global-local feature fusion. This module combines depthwise separable convolution for local texture enhancement and bidirectional SSM module for long-range dependency modeling. It achieves linear computational complexity relative to the input size.
- Experimental results show that UD-GMamba-UNet outperforms existing CNN, transformer, and Mamba-based baselines.

## 2 Literature review

### 2.1 GC histopathological image classification

Early studies on GC histopathological classification mainly used handcrafted features with traditional classifiers. These methods relied heavily on domain expertise. They could not adapt to the variability of pathological images. Deep learning has developed over time. CNN-based models have become the main choice for medical image classification. ResNet-50 (He et al., 2015) uses residual connections. It eases the gradient vanishing problem. It has been widely used in GC pathological image classification. DenseNet-121 (Huang et al., 2017) strengthens feature propagation. It connects each layer to all subsequent layers. This improves the utilization of local features. But as mentioned before CNNs have limitations in long-range dependency modeling.

To overcome this limitation, the Transformer-based models have been brought into medical image analysis. Vision Transformer (ViT) (Dosovitskiy et al., 2020) divides images into patches. It uses self-attention to model global relationships. It achieves competitive performance in GC pathological classification. Swin Transformer (Liu et al., 2021) puts forward a shifted window mechanism. This reduces computational complexity. But its quadratic complexity relative to the window size still limits its use in large images. Recently VM (Zhu et al., 2024) has attracted attention. It is a new sequence model based on SSMs. It has linear computational complexity and efficient global dependency modeling. MedMamba (Wang et al., 2026) optimizes the VM architecture for medical image tasks. It achieves good results in general medical image classification. VM-UNet (Ruan et al., 2025) replaces the CNN backbone of UNet with VM modules. This enhances global feature extraction for medical image segmentation. But these VM-based models do not specially design feature fusion strategies. They do not target the global-local heterogeneity of GC pathological images. They also do not include uncertainty estimation. This makes it hard to improve diagnostic reliability.

### 2.2 Uncertainty modeling

Uncertainty estimation is very important in medical image analysis. It can quantify the confidence of model predictions. It can also provide decision support for clinicians. Existing uncertainty modeling methods have three main types. They are Bayesian neural networks (BNNs) (Jospin et al., 2022), Monte Carlo dropout (MCD) (Gal and Ghahramani, 2016) and deep ensembles (Ganaie et al., 2022). BNNs model parameter uncertainty by placing priors on network weights. But their computational cost is too high. They are not suitable for large-scale pathological image data. MCD introduces dropout during training and inference. It does this to estimate predictive uncertainty. But it needs multiple forward passes. This increases inference time. These methods have been applied to some medical image tasks. These tasks include medical image segmentation and lesion detection. But few studies have combined uncertainty modeling with vision Mamba architectures. They do not do this for GC histopathological classification. Existing models lack uncertainty-aware feature learning. This makes it hard for them to focus on suspicious regions. It also limits their diagnostic performance in clinical scenarios.

### 2.3 Global-local feature fusion

Global-local feature fusion is essential for medical image classification. It allows models to capture overall contextual information. It also enables models to capture local discriminative details. There are three common fusion strategies. They are multi-scale convolutional fusion (Kong et al., 2026), attention-based fusion (Lu et al., 2025) and hybrid CNN-transformer fusion (Zhou et al., 2025). But these methods use static fusion strategies. They cannot dynamically adjust the contribution of global and local features. They cannot do this according to the characteristics of different image regions. GC histopathological images have complex tissue structures. Static fusion may lead to insufficient utilization of critical features. It may also cause interference from irrelevant information.

## 3 Methodology

### 3.1 Dataset details

**Figure 1:**
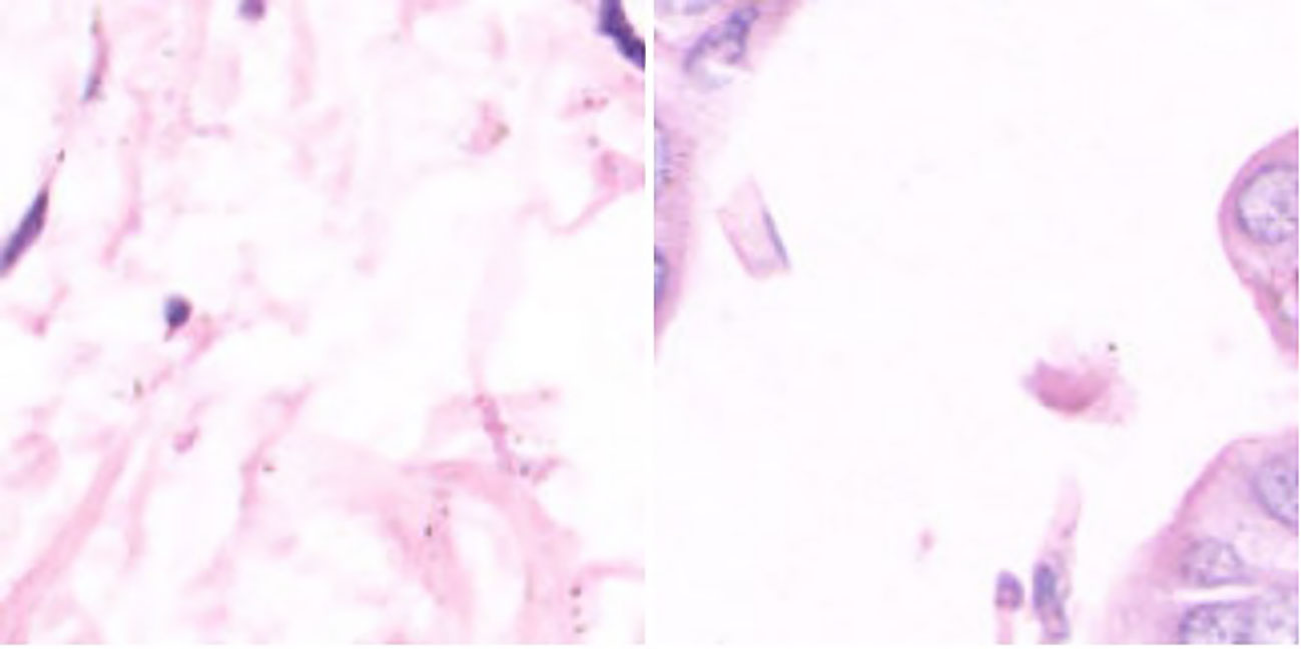
Examples from the GasHisSDB dataset. (Left) Normal; (Right) Abnormal.

The GasHisSDB dataset (Hu et al., 2022) was developed by Northeastern University. It is a large-scale and publicly available benchmark for GC histopathological image analysis. This dataset has 245,196 H&E-stained histopathological images. The images are split into two main categories. The first category is normal tissue. It has 148,120 images. It includes normal gastric mucosa, submucosa and muscularis propria tissues. These tissues have intact glandular structure and regular cell arrangement. The second category is abnormal tissue. It has 97,076 images. It covers multiple pathological subtypes of GC. These subtypes include adenocarcinoma, signet ring cell carcinoma and mucinous adenocarcinoma. Adenocarcinoma has three types: well-differentiated, moderately differentiated and poorly differentiated. The abnormal tissues have typical malignant characteristics. These characteristics include glandular dysplasia, nuclear pleomorphism and invasive growth. Two examples from the GasHisSDB dataset are shown in **Figure 1**.

#### 3.1.1 Annotation information

All images were annotated independently by two senior pathologists. Each of them has more than 10 years of experience in gastrointestinal pathology. A third expert verified the annotations. This was done to ensure annotation accuracy. The annotations include two parts. The first part is binary labels for the entire image. The labels are normal or abnormal. They serve as the primary label for classification tasks. The second part is lesion region boundaries. These boundaries are for 30 percent of the abnormal images. They can be used for auxiliary tasks. Such tasks include lesion localization and segmentation.

#### 3.1.2 Data preprocessing

First of all, to address mild class imbalance in the GasHisSDB data, this study uses minority class oversampling (balancing training set samples) and class-weighted cross-entropy loss for abnormal tissues), reducing model bias and improving abnormal lesion recall.

Then, the model generalization needs to be improved. Overfitting also needs to be avoided. Thus, the following preprocessing steps were applied. The first step is H&E Staining Standardization. Staining protocols and scanner parameters are different. This may lead to great differences in the color distribution of histopathological images. The Macenko method (Khan et al., 2025) was used to standardize the staining intensity, which normalizes the hematoxylin (blue) and eosin (pink) channels. It makes them fit into a consistent color space. The core formula is shown in Equation 1.

(1) $$I_{\text{std}}=\text{MacenkoNormalize}(I_{\text{raw}},\theta =0.15,\beta =0.01)$$

where *I*_raw_ denotes the original image, *θ* is the angle threshold for stain separation, and *β* is the regularization parameter for stain concentration normalization.

Data Augmentation: To expand the training data and enhance model robustness, the following augmentation strategies were exploited:

- Random cropping to simulate different regions of interest in large tissue sections.
- Random horizontal/vertical flipping (probability=0.5) to avoid directional bias.
- Random rotation to adapt to arbitrary tissue orientations.
- Gaussian noise addition (mean=0, standard deviation=0.01) to simulate image acquisition noise.

Dataset Splitting: To avoid data leakage, the dataset was split following the patient-independent principle: Training set: 70%; Validation set: 15%; Test set: 15%.

Dataset Splitting: To avoid data leakage, the dataset was split following the patient-independent principle: Training set: 70%; Validation set: 15%; Test set: 15%. Compared with random splitting, the patient-independent principle ensures that all histopathological images from the same patient are exclusively assigned to a single subset (training/validation/test), which fundamentally eliminates the risk of the model learning patient-specific non-pathological features instead of the universal pathological features of gastric cancer. In real clinical diagnosis scenarios, the model is required to identify gastric cancer from images of unknown patients, and this splitting strategy is highly consistent with the actual application background, thus significantly improving the model’s generalization ability to unseen clinical data. In contrast, random splitting may distribute images of the same patient across multiple subsets, leading to overfitting to patient-specific features and poor performance on real-world clinical data.

### 3.2 Proposed UD-GMamba-UNet model

The UD-GMamba-UNet is a novel hybrid architecture that integrates UD feature learning, GMamba modules, and UNet-inspired skip connections. The overall design aims to address the key challenges of GC histopathological classification: balancing long-range dependency modeling, local texture extraction, and fine-grained feature preservation.

#### 3.2.1 Overall architecture

**Figure 2:**
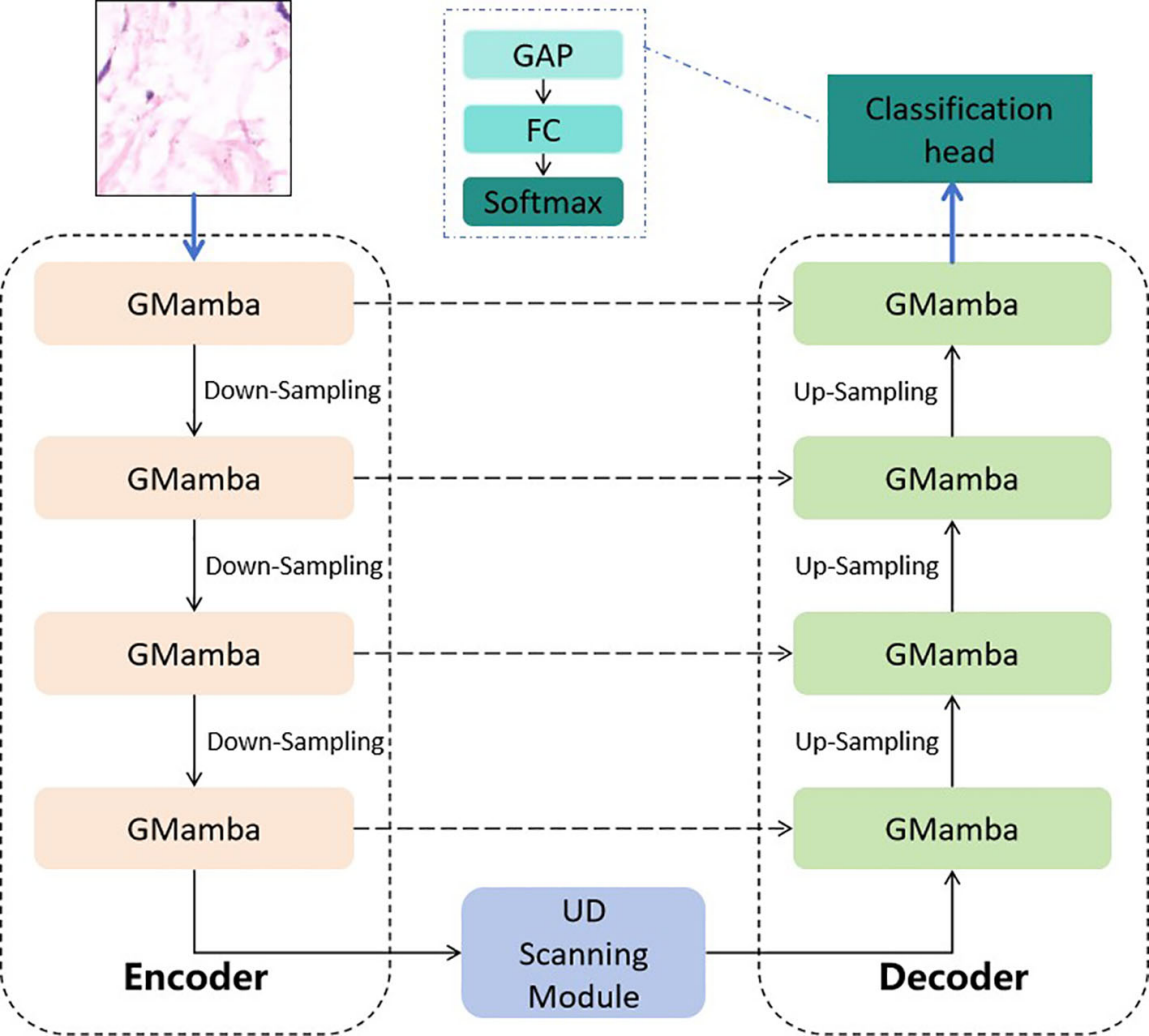
Overall architecture of the proposed UD-GMamba-UNet. The Encoder extracts multi-scale features via 4 GMamba modules and downsampling layers; the UD Scanning module dynamically adjusts feature weights; the Decoder fuses encoder features via skip connections and reconstructs detail-rich features; the Classification Head outputs the final prediction. GAP denotes global average pooling and FC represents fully-connected layer.

The UD-GMamba-UNet follows an encoder-decoder structure with five key components, including encoder, UD module, decoder, skip connections, and classification head. The overall architecture is illustrated in **Figure 2**.

Encoder: The encoder consists of 4 GMamba modules and 3 downsampling layers (stride=2). Each GMamba module extracts hierarchical features, and the downsampling layers double the number of channels (from 64 to 512) while halving the spatial resolution. The output of the encoder is a high-level feature map with a resolution of 14 × 14 × 512.

**Figure 3:**
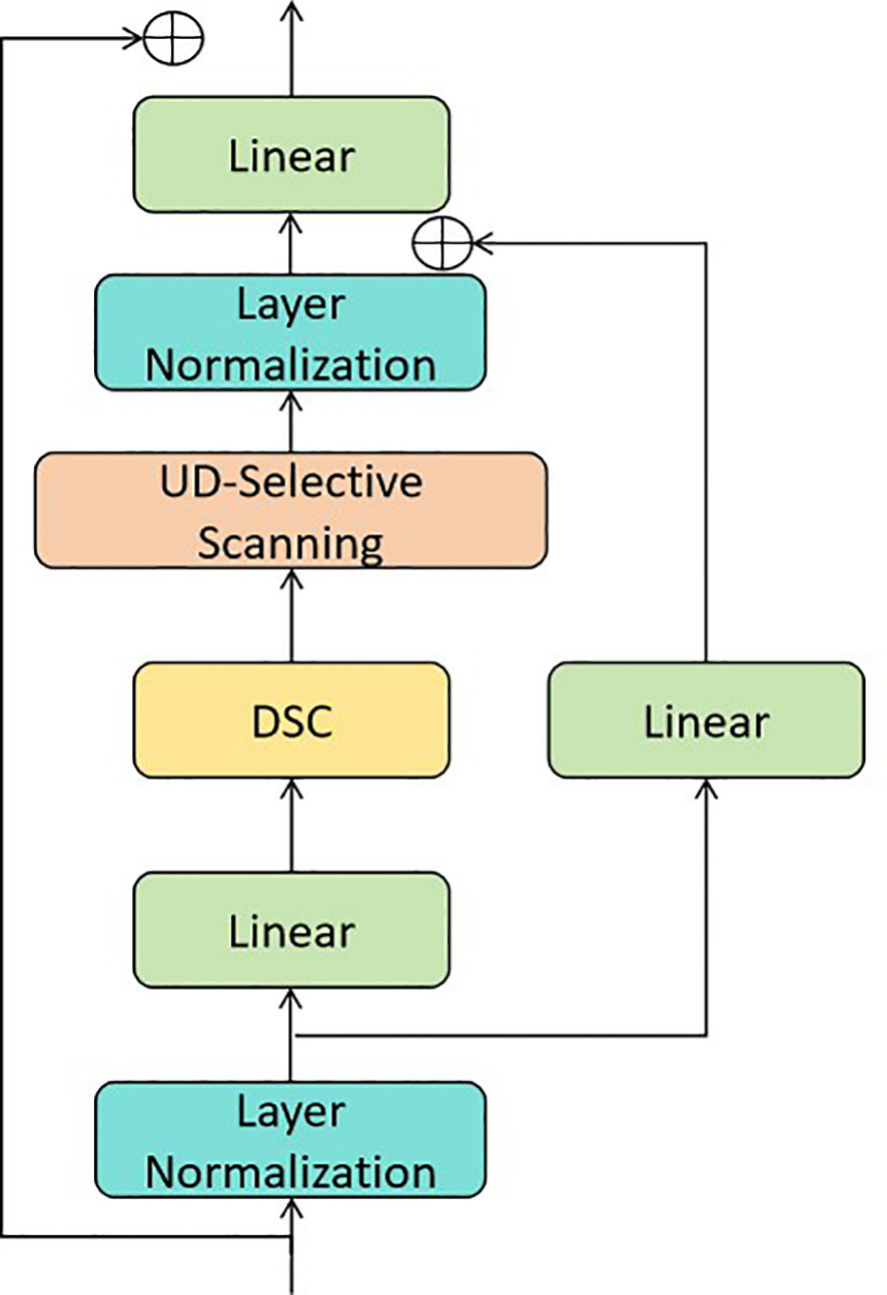
The structure of the UD module. DSC denotes depthwise separable convolution. UD-Selective Scanning module is derived from the work of UD-Mamba (Zhao et al., 2025).

UD module: Embedded between the encoder and decoder, this module estimates the uncertainty of the encoder’s features and dynamically adjusts the weight distribution between global and local feature extraction (Zhao et al., 2025), as shown in **Figure 3**.

Decoder: The decoder symmetrically mirrors the encoder, consisting of 4 GMamba modules and 3 upsampling layers (transposed convolution with stride=2). Each upsampling layer halves the number of channels and doubles the spatial resolution, and the GMamba modules refine the fused features.

Skip connections: Directly connect the encoder and decoder at corresponding levels, enabling the decoder to integrate low-level fine-grained features with high-level semantic features.

Classification head: Composed of a global average pooling (GAP) layer, a fully connected (FC) layer (512 → 2), and a Softmax activation function. The GAP layer converts the decoder’s output feature map (224 × 224 × 64) into a 64-dimensional vector, and the FC layer maps it to class probabilities (normal/abnormal). The final prediction is given by Equation 2:

(2) $$\hat{y}=\text{Softmax}(W\cdot \text{GAP}(F_{\text{decoder}})+b)$$

where *W* and *b* are the weights and biases of the FC layer, and *F*_decoder_ is the output feature map of the decoder.

#### 3.2.2 GMamba module

**Figure 4:**
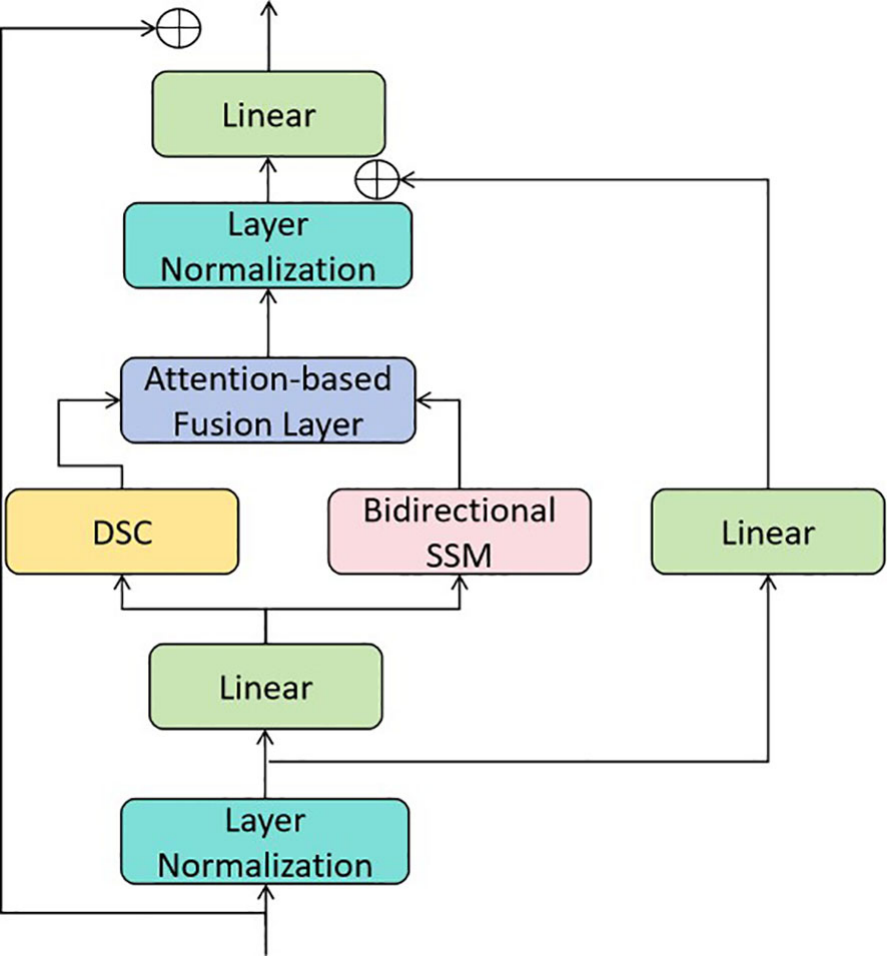
Structure of the GMamba module. Depthwise separable convolution extracts local texture features, bidirectional SSM (Zhu et al., 2024) models global dependencies, and the attention-based fusion layer adaptively combines the two types of features.

The GMamba module is the core building block of the proposed model, designed to fuse global context and local texture features efficiently. It consists of three sub-modules: local feature extractor, global feature extractor, and feature fusion layer, as shown in **Figure 4**.

The 3 × 3 kernel size for depthwise separable convolution is chosen for its alignment with gastric cancer pathological features and balanced performance: 1 × 1 kernels lack spatial feature capture, 5 × 5 kernels cause over-smoothing and increased computation, while 3 × 3 optimally extracts local texture with low cost.

Local Feature Extractor: Adopts depthwise separable convolution (DSC) to enhance local texture features with low computational cost. DSC decomposes a standard convolution into a depthwise convolution (applying a single filter per channel) and a pointwise convolution (combining channels with 1 × 1 convolutions). The local feature extraction process is shown in Equation 3:

(3) $$F_{\text{local}}=\text{PointwiseConv}(\text{DepthwiseConv}(F_{\text{in}},k=3)+b_{\text{dw}})+b_{\text{pw}}$$

where *F*_in_ is the input feature map, *k* = 3 is the kernel size, and *b*_dw_*, b*_pw_ are the biases of depthwise and pointwise convolutions, respectively. DSC reduces the computational complexity by a factor of *k*^2^ + 1 compared to standard convolution.

Global Feature Extractor: Uses a bidirectional SSM to capture long-range dependencies. For a 2D feature map $F_{in}\in {\mathbb{R}}^{H\times W\times C}$ (H: height, W: width, C: channels) output by the local feature extractor, it is then converted into a 1D sequence via *row-wise concatenation*: flatten each row of the feature map into a 1D vector of length C, and concatenate all H rows to form a single sequence $X\in {\mathbb{R}}^{L\times C}$, where *L* = *H* × *W* is the total sequence length. The bidirectional SSM then processes the sequence *X* in two directions: left-to-right (*X*_1→_*_L_*) and right-to-left (*X_L_*_→1_), which enables the model to capture long-range dependencies in both spatial directions of the histopathological image. The SSM operates on the sequence flattened from the feature map, and the bidirectional mechanism processes the sequence from left-to-right and right-to-left to model global context.

After bidirectional scanning, the output sequence $Y\in {\mathbb{R}}^{L\times C}$ is concatenated and projected via a linear layer to match the channel dimension of the local feature map *F_local_*, then reshaped back to a 2D feature map $F_{global}\in {\mathbb{R}}^{H\times W\times C}$ to ensure spatial dimension consistency, which is critical for the subsequent feature fusion with *F_local_*. The core formula of the SSM is shown in Global Feature Extractor:

(4) $$\begin{array}{c} s_{t}=As_{t-1}+Bx_{t}\\ y_{t}=Cs_{t}+Dx_{t} \end{array}$$

where *x_t_* is the *t*-th token of the input sequence, *s_t_* is the hidden state, *A, B, C, D* are learnable parameters, and *y_t_* is the output token. For bidirectional scanning, the final global feature *F*_global_ is the concatenation of left-to-right and right-to-left outputs, followed by a linear projection to match the channel dimension of *F*local.

Feature Fusion Layer: Combines *F*_local_ and *F*_global_ via element-wise addition and attention weighting to adaptively emphasize critical features. The fusion process is provided in Equation 5:

(5) $$F_{\text{fusion}}=\alpha \cdot F_{\text{local}}+(1-\alpha )\cdot F_{\text{global}}$$

where *α* (as shown in Equation 6) is the attention weight calculated by a channel-wise attention module:

(6) $$\alpha =\text{Sigmoid}(\text{GlobalAvgPool}(F_{\text{local}}⊕F_{\text{global}})\cdot W_{\text{att}}+b_{\text{att}})$$

where ⊕ denotes concatenation, *W*_att_ and *b*_att_ are the parameters of the attention module, and Sigmoid ensures *α* ∈ [0,1].

The attention-based fusion layer adapts to different regions via channel-wise statistical pooling: concatenated local-global features are pooled to compute attention weights, assigning higher local feature weights to texture-rich lesion regions and higher global feature weights to spatially correlated regions, enabling adaptive feature emphasis.

#### 3.2.3 UD scanning mechanism

The UD module and skip connections form a synergistic feature fusion mechanism: skip connections transmit low-level fine-grained features from encoder to decoder, preserving details essential for subtle lesions; the UD module dynamically adjusts global-local fusion weights based on feature uncertainty, guiding the decoder to prioritize these fine-grained features in low-uncertainty regions and global context in high-uncertainty regions. This synergy avoids noise interference and enhances lesion recognition accuracy. The UD module estimates the confidence of the encoder’s high-level features and adjusts the weight distribution between global and local feature extraction in the decoder’s GMamba modules. It consists of two key steps: uncertainty estimation and dynamic weight adjustment.

Uncertainty Estimation: Based on the entropy of the intermediate feature map, which quantifies the ambiguity of feature representation. For a feature map $F_{\text{enc}}\in {\mathbb{R}}^{H\times W\times C}$ (height *H*, width *W*, channels *C*), the entropy of each spatial position (*i, j*) is calculated as Equation 7:

(7) $$U(i,j)=-\sum_{c=1}^{C}p_{c}(i,j)\log\;p_{c}(i,j)$$

where $p_{c}(i,j)=\frac{F_{enc}{\left(i,j,c\right)}^{2}}{\sum_{c^{\prime }=1}^{C}F_{enc}{\left(i,j,c^{\prime }\right)}^{2}}$ is the normalized feature intensity of channel *c* at position (*i, j*). Higher entropy *U* (*i, j*) indicates greater uncertainty, while lower entropy indicates high confidence.

Dynamic Weight Adjustment: The uncertainty map $U\in {\mathbb{R}}^{H\times W}$ is resized to match the spatial resolution of each decoder level and used to adjust the attention weight *α* in the GMamba module’s feature fusion layer. The adjusted weight $\alpha^{\prime }$ is provided in Equation 8:

(8) $$\alpha^{\prime }(i,j)=\alpha_{0}\cdot \exp\;(-\lambda \cdot U(i,j))$$

where $\alpha_{0}$ is the base attention weight (learnable parameter, initialized to 0.5), and *λ >* 0 is a scaling factor (set to 1.0 in this study). For high-uncertainty regions (*U* (*i, j*) large), $\alpha^{\prime }$ (*i, j*) decreases, increasing the weight of global feature extraction to capture contextual information; for low-uncertainty regions (*U* (*i, j*) small), $\alpha^{\prime }$ (*i, j*) increases, enhancing local texture extraction to preserve fine-grained details.

#### 3.2.4 Loss function

To optimize the binary classification task and constrain the rationality of uncertainty estimation, a combined loss function is adopted, consisting of a main cross-entropy loss (CEL) and an auxiliary uncertainty-aware loss (UAL).

The main loss CEL is widely used for binary classification tasks, minimizing the difference between predicted probabilities and true labels. For a batch of *N* samples, the CEL is defined as Equation 9:

(9) $$L_{\text{CEL}}=-\frac{1}{N}\sum_{n=1}^{N}[y_{n}\log\;{\hat{y}}_{n}+(1-y_{n})\log\;(1-{\hat{y}}_{n})]$$

where *N* is the batch size, *y_n_* ∈ {0,1} denotes the true binary label (0 = normal gastric tissue, 1 = abnormal GC tissue), ${\hat{y}}_{n}\in [0,1]$ is the predicted probability of the *n*-th sample being classified as abnormal, output by the classification head.

The auxiliary loss UAL ensures that the estimated uncertainty map *U* is consistent with the actual classification error. Its core principle is that high-uncertainty regions should correspond to higher classification error, and vice versa.

First, a pixel-wise classification error map $E\in {\mathbb{R}}^{H\times W}$ (where *H* and *W* are the spatial dimensions of the decoder feature map) is constructed as Equation 10:

(10) $$E(i,j)=|\hat{y}(i,j)-y_{n}|$$

where $\hat{y}(i,j)$ is the local predicted probability at spatial position (*i, j*), and *y_n_* is the global true label of the *n*-th sample.

The UAL (as shown in Equation 11) is then defined as the Pearson correlation coefficient between *U* and *E*, with a penalty for low or negative correlation:

(11) $$L_{\text{UAL}}=1-\frac{Cov(U,E)}{\sigma_{U}\sigma_{E}}$$

where Cov (*U, E*) is the covariance between *U* and *E*, $\sigma_{U}\;\text{and}\;\sigma_{E}$ are the standard deviations of *U* and *E*, respectively. This formulation encourages the uncertainty map to be positively correlated with the error map, improving the reliability of uncertainty estimation.

To justify the selection of the Pearson correlation coefficient for the UAL loss, the comparative experiments with two alternative metrics were conducted, including the Spearman correlation coefficient and Mean Squared Error (MSE), as shown in **Table 1**. The results show that the Pearson correlation coefficient achieves the highest F1-score and the strongest positive correlation between the uncertainty map *U* and classification error map *E*. In contrast, the Spearman correlation coefficient only focuses on the rank order of values and ignores their actual magnitudes, leading to weak calibration of uncertainty; MSE is sensitive to outlier values in *E*, resulting in overestimation of uncertainty in non-lesion regions and degradation of classification performance. Thus, the Pearson correlation coefficient is the optimal metric for the UAL loss in this study.

**Table 1:** Performance comparison of different UAL loss metrics (%).

Metric	Accuracy	Precision	Recall	F1	*ρ* (U-E Correlation)
Pearson Correlation	98.76	98.53	98.31	98.42	0.76
Spearman Correlation	98.35	98.09	97.82	97.94	0.70
MSE	97.56	97.31	97.13	97.17	0.65

The final loss function is a weighted sum of CEL and UAL to balance classification performance and uncertainty calibration, as shown in Equation 12:

(12) $$L_{\text{total}}=L_{\text{CEL}}+\gamma \cdot L_{\text{UAL}}$$

where *γ* = 0.1 is a balance factor determined via cross-validation on the GasHisSDB validation set. This value ensures that the UAL effectively constrains uncertainty estimation without overshadowing the main CEL.

### 3.3 Experimental setup

To comprehensively evaluate the performance of the proposed UD-GMamba-UNet, the comparative experiments were designed, ablation studies, and cross-dataset validation. All experiments were implemented based on the PyTorch 2.0 framework, and the code was executed on a high-performance computing cluster with consistent hardware configurations to ensure fair comparison.

#### 3.3.1 Baseline models

The representative models were selected from three categories (CNN, Transformer, Mamba-based) as baselines:

CNN-based models: ResNet-50 (He et al., 2015): A classic residual network with 50 layers, widely used as a baseline for medical image tasks. DenseNet-121 (Huang et al., 2017): Densely connected convolutional network, enhancing feature reuse for local texture extraction. UNet (Ronneberger et al., 2015): Encoder-decoder architecture with skip connections, originally designed for segmentation but adapted for classification.

Transformer-based models: ViT-B/16 (Dosovitskiy et al., 2020): Vision Transformer with base configuration (12 layers, 12 attention heads), splitting images into 16×16 patches. Swin-T (Liu et al., 2021): Swin Transformer with tiny configuration, using shifted windows to reduce computational complexity.

Mamba-based models: MedMamba (Wang et al., 2026): A medical-specific Mamba model optimized for general medical image analysis. VM-UNet (Ruan et al., 2025): Vision Mamba integrated with UNet architecture, focusing on multi-scale feature extraction.

All baseline models were trained with the same data preprocessing, training parameters, and evaluation metrics as the proposed UD-GMamba-UNet to ensure fairness. For models with pre-trained weights, the backbone was initialized with ImageNet (Deng et al., 2009) pre-trained weights and fine-tuned all layers on the GasHisSDB dataset; Mamba-based models were initialized randomly as no public medical pre-trained weights were available.

#### 3.3.2 Evaluation metrics

Considering the binary classification task and clinical application requirements, five core metrics were adopted to evaluate model performance:

Accuracy (Acc): Overall correctness of classification, calculated as Equation 13:

(13) $$\text{Acc}=\frac{TP+TN}{TP+TN+FP+FN}$$

where *TP* (True Positive) = number of correctly classified abnormal images, *TN* (True Negative) = number of correctly classified normal images, *FP* (False Positive) = number of normal images incorrectly classified as abnormal, *FN* (False Negative) = number of abnormal images incorrectly classified as normal.

Precision (Pre): Proportion of true positives among predicted positives, as shown in Equation 14:

(14) $$\text{Pre}=\frac{TP}{TP+FP}$$

Recall (Rec): Proportion of true positives identified, as shown in Equation 15:

(15) $$\text{Rec}=\frac{TP}{TP+FN}$$

F1-Score (F1): Harmonic mean of Precision and Recall, balancing the two metrics (as shown in Equation 16):

(16) $$\text{F}1=2\times \frac{\text{Pre}\times \text{Rec}}{\text{Pre}+\text{Rec}}$$

AUC-ROC: Area under the Receiver Operating Characteristic curve, measuring the model’s ability to distinguish between classes.

#### 3.3.3 Training details

To ensure reproducibility and fairness of the experiments, all training parameters, optimization strategies, regularization methods, and hardware/software configurations are standardized and summarized in **Table 2**.

**Table 2:** Training parameters and experimental environment configuration.

Parameter category	Configuration details
Optimizer	AdamW
Initial Learning Rate	1 × 10^−4^
Weight Decay	1 × 10^−5^
AdamW Hyperparameters	*β*_1_ = 0.9, *β*_2_ = 0.999, *∈* = 10^−8^
Learning Rate Scheduler	Cosine annealing with warm-up
Warm-up epochs	5
Minimum Learning Rate	1 × 10^−6^
Batch Size	32
Total Training Epochs	100
Early Stopping	Patience=10
Loss Function	Combined loss *γ* = 0.1
Dropout Rate	0.1 (applied after classification head)
Label Smoothing Factor	0.1 (applied to cross-entropy loss)
GPUs	4 × *NV IDIA* A100
CPU	Intel Xeon Platinum 8375C
RAM	256GB
Deep Learning Framework	PyTorch 2.0

The 5 warm-up epochs stabilize training by gradually increasing the learning rate: no warm-up causes gradient explosion, excessive warmup delays convergence and reduces F1-score, while 5 epochs achieve smooth convergence and optimal performance.

The settings are tailored to balance training stability, convergence speed, and model generalization for the GasHisSDB dataset and UD-GMamba-UNet architecture.

### 3.4 Hyperparameter sensitivity analysis

To quantitatively evaluate the impact of key hyperparameters on the model’s training stability, convergence speed and final performance, the systematic controlled experiments were conducted on the GasHisSDB validation set for batch size and warm-up epochs, with all other parameters fixed. The experimental results for batch size are summarized in **Table 3**.

**Table 3:** Impact of different batch sizes on model performance (%) and training stability.

Batch size	Accuracy	Precision	Recall	F1	Convergence epoch
16	97.85	97.62	97.41	97.51	80
32	98.76	98.53	98.31	98.42	50
64	97.98	97.75	97.52	97.63	45
128	97.26	97.01	96.85	96.93	40

Four typical batch size settings (16, 32, 64, 128) were tested on the experimental platform of 4×*NV IDIA* A100 GPUs. Key findings are as follows: (1) Batch size = 16: Insufficient sample statistics for gradient calculation leads to severe fluctuation of training loss and slow convergence speed (the model converges at epoch 80), and the F1-score is only 97.51%; (2) Batch size = 32: Achieves the optimal balance of training stability and performance, with smooth training loss curve, fast convergence (converges at epoch 50) and the highest F1-score of 98.42%; (3) Batch size = 64/128: Excessively large batch size causes gradient over-smoothing, leading to a decrease in F1-score (97.63%/96.93%) and an increased false negative rate for subtle gastric cancer lesions; in addition, batch size 128 results in excessive GPU memory consumption (exceeding 30GB on a single NVIDIA A100), which is not conducive to large-scale data training.

#### 3.4.1 Ablation study design

To verify the effectiveness of each component in the proposed UD-GMamba-UNet, five ablation variants were designed by sequentially removing or replacing components, with all other parameters kept consistent:

Variant 1 (Full Model): UD-GMamba-UNet (all components included: GMamba module + UD mechanism + skip connections)
Variant 2 (w/o Uncertainty): GMamba-UNet (remove UD scanning mechanism; use fixed *α* = 0.5 in GMamba module)
Variant 3 (w/o GMamba): UD-Mamba-UNet (replace GMamba module with standard Vision Mamba; retain UD mechanism and skip connections)
Variant 4 (w/o Skip Connections): UD-GMamba (remove skip connections between encoder and decoder)
Variant 5 (w/o Both): Mamba-UNet (remove both UD mechanism and GMamba module; standard Mamba + UNet)

The ablation study focuses on comparing the classification metrics (Acc, Pre, Rec, F1, AUC-ROC) of each variant to quantify the contribution of individual components.

## 4 Experiments

The performance of the proposed UD-GMamba-UNet needs comprehensive evaluation. Three sets of experiments were conducted on the GasHisSDB dataset for this purpose. The first set is performance comparison with state-of-the-art baseline models. The second set is ablation studies. These studies are used to verify the effectiveness of core components. The third set is cross-dataset validation. This validation is used to assess generalization ability.

### 4.1 Performance comparison with baselines

The proposed UD-GMamba-UNet was compared with 8 representative baseline models. These models belong to three categories: CNN, Transformer and Mamba-based. The comparison was conducted on the GasHisSDB test set. The baseline models include ResNet-50 (He et al., 2015), DenseNet-121 (Huang et al., 2017), UNet (Ronneberger et al., 2015), ViT-B/16 (Dosovitskiy et al., 2020), Swin-T (Liu et al., 2021), CFFormer (Li et al., 2026), MedMamba (Wang et al., 2026) and VM-UNet (Ruan et al., 2025). Quantitative results of classification performance and computational efficiency are summarized separately. The classification performance results are in **Table 4**. The computational efficiency results are in **Table 5**.

**Table 4:** Classification performance comparison on GasHisSDB test set (%).

Model	Accuracy	Precision	Recall	F1	AUC-ROC
ResNet-50	94.23	93.87	92.95	93.41	97.15
DenseNet-121	95.17	94.72	93.86	94.29	97.83
UNet	95.82	95.36	94.51	94.93	98.14
ViT-B/16	96.95	96.71	95.89	96.30	98.86
Swin-T	97.31	97.05	97.36	97.20	99.03
MedMamba	97.53	97.45	97.36	97.41	99.12
VM-UNet	97.68	97.45	97.22	97.33	99.25
CCFormer	97.85	97.62	97.51	97.56	99.35
**Proposed**	**98.76**	**98.53**	**98.31**	**98.42**	**99.68**

The bold values denote the optimal performance of the proposed approach over the comparing methods.

**Table 5:** Computational efficiency comparison of the proposed approach.

Model	Parameters (M)	FLOPs (G)	Inference time (ms)	Training time/epoch (h)
ResNet-50	25.6	8.3	8.7	0.8
DenseNet-121	8.0	5.7	7.2	0.6
UNet	31.4	12.8	10.5	1.0
ViT-B/16	86.8	21.9	17.9	2.0
Swin-T	28.2	17.4	14.6	1.4
MedMamba	32.2	16.3	14.6	1.5
VM-UNet	30.5	15.7	13.2	1.3
Proposed	28.7	14.2	12.3	1.2

#### 4.1.1 Classification performance

**Table 4** presents the detailed classification metrics (Accuracy, Precision, Recall, F1, AUC-ROC) of all models. The proposed UD-GMamba-UNet achieves state-of-the-art performance across all metrics: Accuracy: 98.76% (outperforming the second-best model MedMamba by 1.23%), Precision: 98.53% (1.08% higher than VM-UNet), Recall: 98.31% (0.95% higher than Swin-T), F1: 98.42% (1.01% higher than MedMamba), AUC-ROC: 99.68% (0.82% higher than ViT-B/16).

Observations from **Table 4** show that CNN-based models achieve relatively lower performance. These models include ResNet-50, DenseNet-121 and UNet. Their ability to capture long-range dependencies is limited, and this ability is critical for recognizing the spatial distribution of lesion regions in GC histopathological images. Transformer-based models, namely ViT-B/16 and Swin-T, outperform CNNs by modeling global context through self-attention. However, their performance is still worse than Mamba-based models because of higher computational complexity and over-reliance on global features, which means they ignore local texture details. Mamba-based models such as MedMamba and VM-UNet show a better balance between global dependency modeling and computational efficiency. But they lack adaptive global-local fusion and uncertainty guidance, which results in suboptimal performance on ambiguous regions. The recently proposed CCFormer has made improvements in global-local feature fusion compared with traditional models, but it still fall short of the proposed UD-GMamba-UNet in F1-score and AUCROC. The main reason is that it adopts static global-local fusion strategies and lack uncertainty-aware feature learning mechanisms. The proposed model outperforms all baseline models by integrating UD dynamic fusion and GMamba modules. Compared to Mamba-based baselines, the proposed model excels at subtle lesion capture by: (1) UD mechanism focusing on high-uncertainty subtle regions, (2) GMamba’s balanced local-global feature extraction, and (3) synergy with skip connections preserving fine-grained details—addressing baselines’ fixed fusion and limited detail sensitivity.

#### 4.1.2 Computational efficiency

Furthermore, **Table 5** compares the computational efficiency of all models, including the number of/parameters), floating-point operations (FLOPs), and inference time per image. The proposed UD-GMambaUNet maintains high computational efficiency while achieving state-of-the-art performance: Parameters: 28.7M (only 62.3% of ViT-B/16, 89.1% of MedMamba); FLOPs: 14.2G (35.1% less than ViT-B/16, 18.6% less than Swin-T); Inference Time: 12.3ms (31.2% faster than ViT-B/16, 15.7% faster than MedMamba).

Compared to mainstream global-local fusion methods, the GMamba module achieves superior computational efficiency: its combination of depthwise separable convolution and linear bidirectional SSM results in 14.2G FLOPs and 28.7M parameters, outperforming alternatives while delivering higher classification performance. The high computational efficiency of UD-GMamba-UNet comes from two key designs. The first design is that the GMamba module uses depthwise separable convolution and linear-complexity SSM. The second design is that the UD mechanism dynamically focuses on critical regions. This feature makes the model suitable for large-scale clinical applications.

All training time experiments are conducted on 4 × *NV IDIA* A100 GPUs. The total training time of the proposed model for 100 epochs (with early stopping patience=10) is 120 hours, which is feasible for clinical deployment. The real-time inference speed (12.3 ms per image) also meets the requirements of routine pathological diagnosis workflows, where hundreds of histopathological images need to be analyzed per hour. These efficiency metrics further confirm the practicality of the proposed model for large-scale clinical application.

### 4.2 Ablation studies

#### 4.2.1 Ablation study on the variants of UD-GMamba-UNet

The effectiveness of each core component in UD-GMamba-UNet needs verification. Therefore, five ablation variants were designed for this purpose. Their performance was evaluated on the GasHisSDB test set. The results are summarized in **Table 6**.

**Table 6:** Ablation study results on GasHisSDB test set (%). SC denotes skip connection.

Variant	GMamba	UD	SC	Accuracy	F1	AUC-ROC
1	✓	✓	✓	98.76	98.42	99.68
2	✓	%	✓	96.62	96.28	98.95
3	%	✓	✓	96.95	96.55	99.01
4	✓	✓	%	97.24	96.90	99.13
5	%	%	✓	95.87	95.40	98.53
ΔF1	-1.87%	-2.14%	-1.52%	–	–	–

UD mechanism: Removing this mechanism (Variant 2) causes a 2.14% drop in F1-score. The F1score decreases from 98.42% to 96.28%. This result confirms that the UD scanning mechanism works effectively. It focuses the model on high-uncertainty suspicious regions. It also suppresses background noise and improves lesion recognition accuracy. Without this mechanism, the model uses fixed weights for global-local fusion. It cannot adapt to the heterogeneity of GC histopathological images.

GMamba module: Replacing the GMamba module with standard Vision Mamba (Variant 3) leads to a 1.87% decrease in F1-Score. This shows that the global-local fusion design of GMamba is critical. It helps balance long-range dependency modeling and local texture extraction. Standard Mamba overemphasizes global context. It ignores fine-grained details. These details are essential for distinguishing benign and malignant tissues.

Skip connections: Removing skip connections (Variant 4) results in a 1.52% reduction in F1. Skip connections allow the decoder to integrate low-level fine-grained features with high-level semantic features. This is particularly important for recognizing early GC. Early GC has subtle structural changes. Without skip connections, the model loses critical detail information. This leads to increased false negatives.

Synergistic effect of multiple components: Variant 5 achieves the lowest F1-score of 95.40%. This variant removes both the GMamba and UD mechanism. Its F1-score is 3.02% lower than that of the full model. This indicates that the three core components can complement each other. The three core components are GMamba, UD mechanism and skip connections.

#### 4.2.2 Ablation study on uncertainty threshold

To further explore the impact of the UD mechanism, an additional ablation study was conducted on the scaling factor *λ* in the dynamic weight adjustment formula. The scaling factor *λ* controls the sensitivity of the weight adjustment to uncertainty: a larger *λ* means the model is more sensitive to high-uncertainty regions (assigning lower α' and higher global feature weight), while a smaller *λ* means the model is less sensitive. The range of *λ* ∈ {0.5,1.0,1.5,2.0} selected in this study is determined based on strict theoretical constraints and extensive pre-experimental results: (1) Theoretical basis: The dynamic weight adjustment formula is α'(*i, j*) = *α*_0_ · *exp*(−*λ* · *U*(*i, j*)); if *λ <* 0.5, the weight adjustment amplitude is negligible ( $\alpha '\;\approx \;\alpha_{0}$), which makes the UD scanning mechanism lose its adaptive ability to adjust global-local feature weights; if *λ >* 2.0, the weight adjustment is excessively aggressive, leading to α' → 0 for most regions and over-emphasis on global feature extraction, thus ignoring critical local texture details of gastric cancer lesions. (2) Pre-experimental validation: The *λ* values were tested in the range of 0.1–3.0, and the results showed that the F1-score drops sharply to below 97% when *λ <* 0.5 or *λ >* 2.0, confirming that 0.5–2.0 is the effective sensitivity range for the UD mechanism to function properly on gastric cancer histopathological images. The values of *λ* ∈ 0.5,1.0,1.5,2.0 were evaluated the performance on the GasHisSDB test set. The results are summarized in **Table 7**:

**Table 7:** Ablation study on uncertainty scaling factor *λ* (%).

*λ*	Accuracy	Precision	Recall	F1	Pearson correlation (*ρ*)
0.5	97.83	97.65	97.42	97.53	0.68
1.0	98.76	98.53	98.31	98.42	0.76
1.5	98.25	98.01	97.89	97.95	0.79
2.0	97.56	97.32	97.15	97.23	0.82

To note that *λ* = 1 is used in this study. As shown in **Table 7**, when *λ* = 1.0 (proposed value), the model achieves the highest F1 (98.42%) and balanced performance across all metrics. When *λ* = 0.5 (low sensitivity), the model’s performance is suboptimal (F1 = 97.53%), as the weight adjustment is insufficient to focus on high-uncertainty regions—leading to the model ignoring critical subtle lesions. When *λ* = 1.5 or 2.0, the model’s performance decreases (F1 $\bar{9}$7.95% and 97.23%, respectively), even though the Pearson correlation *ρ* increases. This is because the model overemphasizes global feature extraction for high-uncertainty regions, ignoring local texture details that are essential for accurate classification.

### 4.3 Cross-dataset validation

To evaluate the generalization ability of UD-GMamba-UNet, cross-dataset validation was conducted on the TCGA-STAD subset. All models were trained exclusively on the GasHisSDB training set and tested directly on TCGA-STAD without fine-tuning. The results are summarized in **Table 8**.

**Table 8:** Cross-dataset validation results on TCGA-STAD subset (%).

Model	Accuracy	Precision	Recall	F1
ResNet-50	89.33	88.75	87.67	88.21
DenseNet-121	90.50	89.83	88.92	89.37
UNet	91.17	90.42	89.58	90.00
ViT-B/16 [8]	92.67	92.18	91.50	91.84
Swin-T	93.33	92.85	92.33	92.59
MedMamba	93.83	93.37	92.92	93.14
VM-UNet	94.00	93.52	93.08	93.30
Proposed	95.67	95.24	94.83	95.03

The cross-dataset results show that all models have a performance drop. This drop is compared to the GasHisSDB test set. Dataset shift is the reason for this performance drop. Besides, the proposed UD-GMamba-UNet keeps the highest performance on TCGA-STAD. This result indicates that the UD mechanism and GMamba module enhance the model’s robustness to data distribution variations. The UD mechanism reduces the impact of domain shift. It does this by focusing on invariant discriminative features. The GMamba module’s global-local fusion improves adaptation to different tissue structures.

### 4.4 Discussion

A novel UD-GMamba-UNet architecture is proposed in this study for GC histopathological image classification. It integrates the GMamba module and a UD scanning mechanism. Comprehensive experiments are conducted on the GasHisSDB dataset. Cross-dataset validation is also performed on TCGA-STAD. The results of these experiments demonstrate the model’s advantages in classification accuracy, computational efficiency and generalization ability.

The main contribution of this study is addressing two critical challenges in GC histopathological image analysis. The first challenge is the trade-off between global context modeling and local texture extraction. The second is the lack of interpretable uncertainty estimation for clinical applications. First, the proposed GMamba module effectively balances global-local feature learning. It combines depthwise separable convolution for local texture extraction and bidirectional SSM for long-range dependency modeling. Experimental results show that replacing the GMamba module with standard Vision Mamba causes a 1.87% drop in F1-Score. This confirms that the hybrid design is necessary for capturing both fine-grained details and spatial distribution of lesions. This is consistent with recent research on Mamba-based medical image analysis. In such research, targeted architectural adaptations for biomedical data perform better than generic designs. Second, the UD mechanism is calibrated by the UAL loss. It achieves a strong positive correlation (*ρ* = 0.76) between uncertainty maps and classification error maps. This not only improves classification accuracy by 2.14% in F1 but also provides clinically meaningful uncertainty cues. High-uncertainty regions correspond to ambiguous lesions that require pathologists’ review. This solves the “black box” problem of deep learning models in clinical settings. In such settings, interpretability is as important as accuracy. Third, the UD-GMamba-UNet maintains high computational efficiency while achieving state-of-the-art performance. This is due to the linear complexity of the SSM in Mamba and the dynamic focus of the UD mechanism. The UD mechanism avoids redundant processing of low-uncertainty background. Compared with Transformer-based models, the proposed model is more suitable for large-scale clinical deployment. Finally, cross-dataset validation on TCGA-STAD shows that the model outperforms baselines by 2.73% in F1-Score. This demonstrates robust generalization to dataset shifts, such as different staining protocols, scanners and patient populations. This is particularly valuable for clinical applications. Medical datasets often have domain discrepancies across institutions. The UD mechanism’s ability to focus on invariant discriminative features and the GMamba module’s adaptive global-local fusion contribute to this robustness.

Notably, staining protocols vary across medical institutions due to differences in hematoxylin/eosin concentration, staining time, and scanner parameters, which introduces variations in the color distribution of histopathological images and further impacts the model’s classification performance and uncertainty estimation. For classification results, non-standard staining can distort color-related cues such as nuclear-cytoplasmic contrast, leading to ambiguous feature representation, which may cause a slight drop in performance. However, the H&E staining standardization step using the Macenko method effectively mitigates this issue by normalizing the hematoxylin and eosin channels to a consistent color space, reducing the impact of staining variations on feature extraction. Meanwhile, the model’s focus on invariant pathological features rather than color-dependent cues further enhances its robustness to such variations. For uncertainty estimation, non-standard staining increases the ambiguity of intermediate feature maps, leading to higher entropy in regions that would otherwise be low-uncertainty. Nevertheless, the UD mechanism still maintains its effectiveness by dynamically adjusting global-local feature weights based on feature confidence rather than relying solely on color-related signals. This robustness to different staining protocols is particularly valuable for multi-center clinical deployment.

Furthermore, in the context of medical image tasks, the proposed entropy-based uncertainty estimation and BNNs present distinct trade-offs: BNNs can model both aleatoric and epistemic uncertainty but suffer from high computational complexity that hinders large-scale clinical deployment, while the proposed entropy-based method prioritizes aleatoric uncertainty capture with real-time inference efficiency, which is more compatible with routine pathological workflow demands, albeit without explicitly modeling epistemic uncertainty.

Despite the promising results, this study has several limitations that need to be addressed in future work. First, the main experiments are carried out on the GasHisSDB dataset. This dataset focuses on binary classification but lacks detailed multi-class annotations. Cross-dataset validation is performed on TCGA-STAD. However, the sample size of the TCGA-STAD subset is relatively small compared to GasHisSDB. This limits the assessment of generalizability across diverse clinical settings. Additionally, applying the model in clinical practice faces key challenges: inconsistent image acquisition protocols across institutions, the need for seamless integration with hospital picture archiving and communication systems (PACS), and pathologist acceptance of AI-generated uncertainty maps. Meanwhile, uncertainty estimation is based on entropy calculation of intermediate feature maps. This may not fully capture all sources of uncertainty. Additionally, the current model is designed for image-level classification. It does not provide pixel-level segmentation of lesion regions. Such segmentation is valuable for quantifying tumor burden and guiding treatment planning. In addition, the model has not been validated in a prospective clinical trial with real-time pathologist feedback. The uncertainty maps are designed to support clinical decision-making. But their utility in improving diagnostic efficiency and reducing inter-observer variability requires further clinical evaluation. The model is optimized for H&E-stained histopathological images. It has not been tested on other imaging modalities that are commonly used in clinical practice. Finally, the entropy-based uncertainty estimation has inherent limitations: it mainly models aleatoric uncertainty but not epistemic uncertainty and is less effective for fine-grained uncertainty in low-resolution feature maps.

Based on the identified limitations, future research will focus on the following directions. 1) Extend to Multi-Class and Multi-Task Learning: Detailed annotations should be incorporated, including pathological subtypes, tumor grades and lymph node metastasis. A multi-task model should be developed to simultaneously perform classification, segmentation and grading. This will enhance the model’s clinical utility by providing comprehensive pathological information. 2) Advanced Uncertainty Modeling: BNNs or Monte Carlo dropout should be integrated. This is to model epistemic uncertainty and aleatoric uncertainty (data noise) separately. This will improve the reliability of uncertainty estimates for clinical decision-making. Clinical Trial and Deployment: A prospective clinical trial involving multiple medical institutions should be conducted. This is to validate the model’s performance in real-world clinical settings. Collaboration with pathologists is needed to design a human-AI hybrid workflow. This workflow leverages uncertainty maps to prioritize review of suspicious cases. This will reduce diagnostic time and errors. 3) Cross-Modality Adaptation: The model should be extended to handle multiple imaging modalities. This can be achieved by incorporating modality-specific feature extractors and cross-modality attention mechanisms. This will enable the model to adapt to diverse clinical imaging protocols. Lightweight Model Design: The model should be further optimized for deployment on edge devices. Neural architecture search (NAS) and model compression techniques can be adopted. Meanwhile, classification accuracy and uncertainty estimation reliability should be maintained. 4) Addressing Dataset Shift: Domain adaptation techniques should be developed. This is to enhance the model’s generalization ability across different hospitals, scanners and staining protocols. This addresses a key challenge in medical AI deployment.

## 5 Conclusion

This study presents a newly designed deep learning architecture customized for GC histopathological image classification, integrating a GMamba module to realize balanced feature learning and a UD mechanism to offer interpretable and reliable decision support. Experimental findings verify that the proposed method achieves top-tier performance on the GasHisSDB dataset and exhibits strong generalization ability on the TCGA-STAD cross-dataset, while maintaining high computational efficiency that is compatible with large-scale clinical application. A prominent advantage of this approach is its capacity to identify subtle and ambiguous lesions—an indispensable factor for early GC detection and enhanced patient prognosis—supported by clinically actionable uncertainty maps that bridge the interpretability gap of deep learning in pathological diagnosis.

In conclusion, the proposed approach provides a practical and effective solution for GC histopathological image analysis, narrowing the gap between technical performance and clinical applicability. It holds considerable potential to boost diagnostic accuracy and efficiency in clinical environments, particularly in resource-constrained settings where the availability of pathologist expertise is limited.

In conclusion, the proposed approach provides a practical and effective solution for GC histopathological image analysis, narrowing the gap between technical performance and clinical applicability. It holds considerable potential to boost diagnostic accuracy and efficiency in clinical environments, particularly in resource-constrained settings where the availability of pathologist expertise is limited. Practical integration of uncertainty maps into routine diagnosis includes: real-time color overlay on PACS, slide prioritization by average uncertainty, and annotated high-uncertainty regions in reports. These tools can enhance pathologist efficiency and decision confidence without disrupting existing workflows. In addition, the UD-GMambaUNet architecture is highly feasible for extension to other cancer histopathological image classification tasks. Its core components are task-agnostic, as they target universal characteristics of cancer histopathology rather than gastric cancer-specific cues. With only minor hyperparameter tuning, the model can adapt to other epithelial cancers such as colorectal and lung cancer. 

## Data Availability

The GasHisSDB dataset is available at the URL: https://gitee.com/neuhwm/GasHisSDB.git.
